# Retraction Note to: Workplace bullying and its preventive measures and productivity among emergency department nurses

**DOI:** 10.1186/s13584-019-0330-8

**Published:** 2019-07-04

**Authors:** Suhair Hussni Al-Ghabeesh, Haya Qattom

**Affiliations:** Faculty of Nursing, Head of the Clinical Nursing Department, Al-Zaytoonah University of Jordan, Airport Street, Amman, Jordan

**Retraction Note to: Isr J Health Policy Res (2019) 8: 44. https://doi.org/10.1186/s13584-019-0314-8**

The Publisher has retracted this article [1] because it was published in this journal in error. This article is republished in BMC Health Services Research [2].
